# Treatment of Empyema

**Published:** 1883-08

**Authors:** E. Fletcher Ingals

**Affiliations:** Prof. of Laryngology, Rush Medical College, Prof. of Diseases of the Throat and Chest, Woman’s Medical College, Chicago, Ill.


CHICAGO MEDICAL

JOURNAL & EXAMINER.

VOL. XLVII.—AUGUST, 1883.—NO. 2.

### Original Communications.

ARTICLE I.

TREATMENT OF EMPYEMA. Read before the Chicago Medical Society, April 16, 1883, by E. FLETCHER INGALS, A. M., M. D.,

Prof. of Laryngology, Rush Medical College. Prof. of Diseases of the Throat and Chest, Woman's Medical College, Chicago, Ill.

Empyema, or, as it is often termed, chronic pleurisy, is an affection which has been so long known that little of interest can be said regarding its history, etiology or symptomatology; but much interest must necessarily be felt in the treatment, which has been greatly improved within the past few years.

No longer ago than 1874, Dr. Loomis* wrote that only one-eighth of the cases of empyema which are operated upon recover, while one-fifth of those in which a spontaneous opening occurs get well; but I cannot think there would be such a large mortality if proper treatment was adopted.

Of fourteen cases which have lately come under my observation, where operations have been performed, only five have died,

*Loomis, Diseases of the Respiratory Organs, Heart and Kidneys, p. 200.

and of these, two were moribund when operated on, living only a few hours, while one died three weeks after the operation, apparently from the openiug of an abscess of the liver into the peritoneal sac.

One died three months after the operation from exhaustion. I operated on this case for another physician, and the subsequent treatment was left almost entirely to the patient. Could he have had care, I think he would have recovered.

The fifth case was one in which the effusion was at first sero-purulent, and he died, notwithstanding the best care I could give him.

The two cases that were moribund at the time of aspiration, I had never seen before. I think they might have recovered had they been operated on a few weeks earlier.

Excluding these two cases and the one who died from the opening of the abscess into the peritoneal cavity, only two, who had any reasonable chance of recovery, have died, out of eleven cases, and considering the unfavorable surroundings and lack of treatment of one of these, it seems hardly fair to use it as evidence against the operation.

But one out of ten cases would then be left, and in this the effusion was sero-purulent at first, which condition, according to Bowditch,* is nearly certain to prove fatal. However, at the worst, the deaths were only a fraction over twenty-eight per cent., while the quotation referred to gave over eighty-seven per cent.

Of some of these cases I have no notes; of some, the notes are meager, while others present no features of peculiar interest.

CASE I.—Is interesting from the fact that the patient was only ten months old, and recovered after a single aspiration.

The babe was the daughter of Mr. R., of this city, who called me to see the child on the 10th of last January.

I found her suffering from what appeared to be an ordinary cold, but three or four days later she began to develop signs of pleurisy of the left side.

The disease went on through its usual course until about the end of the third week, after which the patient ceased to improve, and remained in this condition for some time.

*From a personal letter from Bowditch.

She was eating but little, sweating profusely, vomited often and had a bad cough. At the end of the fifth week, finding that there was no tendency toward absorption of the effusion, I aspirated the chest and removed about eleven ounces of pus.

The cavity was not entirely emptied, but as some blood made its appearance, I thought it best not to continue the operation. It is now six weeks since I operated, during which time the patient has steadily improved. There has been no re-filling of the cavity, and the lung has expanded well since I last saw the child, and I have been told by the parents that it appears to be entirely well, having no cough, sweating, indigestion or dyspnœa, and is steadily gaining in weight and strength. *

CASE II.—J. E., æt. thirty-five, came to the dispensary in April, 1881. He gave the history of an acute attack of pleurisy five years previous. I found marked distension of the left side of the chest, flatness on percussion, and absence of respiratory murmur. The heart was crowded two inches to the right, and there was a distinct mitral murmur.

I aspirated the chest and removed three quarts of pus, when I was obliged to stop, because of a sense of pressure on the sternum, though the cavity was not emptied. I repeated the operation six times during the following two months, but was never able to empty the cavity until the last operation. During this time, the fluid had gradually diminished in quantity, and was becoming less purulent in character.

At the seventh operation, I withdrew one quart of slightly purulent fluid, making about twelve and one-half quarts in all. The cavity was then empty, and I determined on a radical operation as soon as it should refill. The patient did not return to me again until the September following, having been working all summer. Upon examination, I found the left side of the chest again distended, measurement showing the circumference of the right and left sides to be respectively sixteen and seven-eighths and seventeen and one-fourth inches. I aspirated, and drew off seventy-four ounces of sero-pus, and two weeks later I removed thirty-six ounces more.

* Since the above writing the father has reported to me that the child is pepfectly well.

The operations were repeated from time to time until October 4, when I introduced the aspirator needle in the axillary region, between the sixth and seventh ribs, and drew off about two ounces, which seemed to empty the cavity. The fluid was slightly tinged with blood, but to the eye had no appearance of pus. This was the thirteenth and last operation.

During the treatment, the patient had taken iodide of potassium, tincture of iron, quinine, strychnine, etc., from time to time, and had been given sedatives when needed, for cough.

I saw the patient again in January, 1883. He had been troubled with a cough for two months, and recently with an abscess in the ear. The side was retracted, and contained no fluid. I ordered a simple cough mixture, which was effective.

I did not see him again until April 3, when he stated that he had been feeling badly for some time, but had been sick only about two weeks. He was coughing considerably, and expectorating mucc-pus. His general appearance was bad. Examining the side, I found it as large as the other, and tympanitic on percussion. Amphoric respiration could be heard over the entire side. There was no fluid in the pleural cavity.

This is one of those rare cases in which repeated aspiration cured the empyema. I should have deemed it best to perform a radical operation in this case early, had I been able to empty the pleural sac without giving pain, but as this was impossible before the seventh operation, on account of compression of the lung of long standing, no other course was left for me. Subsequently, the gradual diminution in the amount of liquid at each operation, and the lessened percentage of pus each time, with its final disappearance, rendered the radical operation unnecessary.

The pneumothorax had been recently developed, and not until all evidence of purulent effusion had been absent for fifteen months. It was doubtless due to rupture of the lung, some part of which had become much weakened by pressure which it had to bear as the result of the retracted condition of the chest wall, which had a constant tendency to regain its former position. Had drainage tubes been inserted in this cavity four or five years earlier, the two surfaces of the pleura would have become adherent, and the pneumothorax would not have occurred.

CASE III.—The notes of the following case have been kindly furnished me by Dr. J. L. Mulfinger, of this city, for whom I operated.

The patient, H. S. a boy, aged three years and nine months, was taken in the latter part of February, 1881, with scarlet fever. The fever ran its usual course, with this exception, that the eruption was not well marked, and lasted but two days. The throat symptoms were unusually severe.

With the subsidence of the rash the temperature gradually diminished until the desquamative stage was reached, at which time it was 100.5° F.

At about this time, although the patient had been closely watched and not allowed to leave the bed, he began to be troubled with a cough, which in a few days became severe, the temperature again rising until it reached 105° F.

The respiration became short and quick, the patient sweating profusely. The doctor found an effusion in the left pleural cavity, and on April 9th I aspirated the chest, drawing off about a quart of pus, when I was obliged to desist on account of the pain. The cavity re-filled, and on April 15th I introduced two drainage tubes, and allowed the fluid to escape until the patient complained of pain. A one per cent. solution of carbolized water was then run into the cavity until it was sufficiently full to relieve the pain. On the following day, the cavity was *thoroughly* washed with carbolized water.

This was repeated twice daily for several days, the tubes being kept closed in the interval. At the end of a week the patient's general condition had so much improved that he was able to eat.

The cavity steadily decreased in size, and in a short time would hold no fluid. The tubes were now gradually withdrawn, the washing out of the cavity being continued, one of the tubes being accidentally washed out about the 5th week.

The other tube was removed a week later, and at the end of the seventh week the patient had completely recovered.

He has since grown well, having pefect use of the lung and no difficulty with the side.

This was the first case in which I used two drainage tubes.

They were inserted through separate openings about an inch apart.

In subsequent operations I used two drainage tubes, but they were both passed through the same opening.

One feature of this case was of special interest as disproving the theory, which I have seen advanced, that there was no need of cleansing the cavity. About five days after the operation, the doctor called, after a longer absence than usual, and found that the patient's temperature was three and one-half degrees higher than when he last saw him. Upon cleansing the pleural sac the temperature fell to its normal point, and did not again rise during the treatment.

CASE IV.—I was called August 1, 1881, in consultation with the late Dr. Mead, to see C. G., who had been taken six or eight weeks previously with an attack of typhoid fever, which had been followed by an attack of pleurisy.

I found the patient in a greatly prostrated condition, and sweating profusely. He was suffering considerable pain, and experiencing great difficulty in breathing, the right side of the chest being distended to a marked degree. The patient was greatly emaciated, and was in a general anasarcous condition. I aspirated the chest, and withdrew about a pint of pus, when I was obliged to desist because of pain. Four days later, I again aspirated, drawing off at this time about twenty-four ounces of pus. On August 8, I performed the radical operation, introducing a large-sized trocar about one and one-half inches to the right of the mammillary line, between the fifth and sixth ribs. Through the canula I inserted two drainage tubes; then withdrawing the canula, the drainage tubes were left in position.

I left instructions for the frequent cleansing of the cavity, and gave directions for the subsequent use of astringent injections. Solutions of sulphate of zinc, varying in strength from gr. ii–xxx to ℥i were used, and later, the sulphate of copper, in solutions varying in strength from gr. v–xx to ℥i. The cavity was washed out twice daily, and part of the time free drainage was allowed by leaving the tubes open, the end resting under water; but much of the time, for the first few weeks, the patient insisted upon keeping the tube closed, as it caused him less inconvenience.

Weak astringent solutions were used at first, the strength being gradually increased, until, at the end of two months, we were using about gr. xx–℥i of the sulphate of copper every other day. The cavity, at this time, had a capacity of about two ounces.

The patient then went into the country, remaining two or three months, during which time he used the injections himself. On his return. I found that the cavity would hold only about ʒii.

In the month of February following, he came to me again, and upon examination, I found the cavity completely obliterated, with the exception of a sinus which extended nearly longitudinally backward from the opening for a distance of about six inches.

To heal this, nitrate of silver in strong solution was used, but with little improvement; afterward tincture of iodine, solid nitrate of silver, solid sulphate of copper, and finally, fuming nitric acid were employed. The latter gave the best results, about one and one-half inches of the sinus being obliterated in a short time, but as the patient was living in the country, so that I could not see him often, I unfor- tunately gave him some of the acid, directing him carefully how to use it, and asking him to come and see me again in three or our weeks.

He used it so faithfully that the former good effects were completely counteracted, and on his return, I found a cavity holding ʒii. Milder measures were then adopted.

My method of using the nitric acid had been to oil a soft rubber catheter thoroughly, and then draw into the distal end of it one or two drops of the acid. The catheter was then introduced into the bottom of the sinus, and the acid expelled by blowing into the tube. The patient, I presume, used five or six drops at a time, and repeated it too often.

Finally, about August 1, I had a long, flexible electrode made, and used it with the galvano-cautery, following the cauterization, for several days, with an injection of a solution of acetate of lead, gr. xx–℥i. The cauterizations were repeated two or three times, with the best of results, the case being reported to me as perfectly cured about a month after the first cauterization.

The electrode was first tested with the battery, to ascertain the length of time necessary for the wire to become red-hot, which was usually about four seconds. After allowing it to cool, it was introduced to the bottom of the sinus, and the electric current turned on; after about five seconds it was withdrawn so as to sear two or three inches of the sinus, and then the current was cut off.

In the five cases remaining which have recovered, there was nothing of peculiar interest.

Of the five patients that died, two, as already stated, were dying at the time of the aspiration, and neither presented any peculiar features. In the two who died of exhaustion after the radical operation, one had good care, and the other practically none.

The remaining one of the fatal cases presented some interesting features, which are detailed below.

CASE V.—The patient, G. T., æt. seventeen, a schoolboy, was taken sick April 22, 1882. He went to bed apparently well, but awakened at 3 A. M. with a severe chill, followed by fever and vomiting, and pain in the right hypochondriac region.

Dr. E. Ingals saw the patient the next day, at which time he seemed much better, having no pain; but about forty-eight hours after the first attack, the pain in the region of the liver again returned, and he was never free from it afterward. Three days later, the abdomen became tympanitic, the pain extending over the entire region, and a few days afterward the pain extended above the diaphragm, the pleura having become involved.

On May 8, after consultation with Dr. E. Ingals, I inserted an aspirator needle near the axillary line, between the fifth and sixth ribs, but obtained no pus. I then inserted it near the angle of the ribs, in the eighth intercostal space, and withdrew seventy-five ounces of pus. The patient now began to complain of pain below the lower border of the ribs on the right side anteriorly, so the aspiration was stopped, but the pain still continued, and became so severe as to cause great depression, and finally collapse, from which he did not rally until after eighteen or twenty hours.

May 14, I introduced two drainage tubes, through a canula, the trocar of which was inserted near the point of former puncture.

Several ounces of pus escaped while the tubes were being introduced, but no air entered. No pus was withdrawn through the tubes. Next day, I found that there had been no leaking, and no bad symptoms. The tubes were opened, but after four ounces had escaped, was obliged to desist on account of the pain.

I saw him again May 20. The tubes had been opened twice a day regularly, and a weak solution of carbolic acid run in at one while the pus escaped at the other, but it was impossible to empty the cavity of the water after the pus had been washed out, on account of the severe pain. The pulse ranged from 104 to 120. Temperature 99.5. For the last two days, the patient had complained much of nausea and "rolling" of the stomach. I then substituted eucalyptol and boracic acid for the carbolic acid.

On the morning of May 25, saw the patient felt and appeared better than ever before since the first operation.

He had been steadily improving until two days previously, since when he had complained rather more than before of pain.

No air had entered the chest, but, although the cavity was evidently much smaller, I was unable to empty it on account of the pain.

In the evening, the father reported to me that the boy had had a chill, and was still cold. I found him in a condition of collapse. When I saw him the next morning, he had not recovered from the collapse, and had vomited frequently a dark greenish fluid.

In the afternoon he was much worse, and appeared to be sinking rapidly.

With each expiration, a large bubble appeared to break through the fluid in the stomach. He died at 6 o'clock the next morning. I could not get a post-mortem.

These cases illustrate what may and what may not be done by operative procedures. The statistics which show that one-fifth of those in whom a spontaneous opening occurred recovered, while only one-eighth of those operated on recovered, would at first seem to render operative procedure unjustifiable; but we must not overlook the fact that in these, the cases which did not live long enough for the opening to occur are not mentioned. If the latter cases could be accurately collected, it would probably be found that not more than one in thirty recover when left to nature.

Four or five operations, similar in character, have been recommended in the treatment of empyema. Of these, aspiration is the first, and should always be done, not only as a means of determining if it be actually empyema, but to ascertain whether or not the cavity may be emptied without causing great pain. The operation itself causes only momentary pain, and the evacuation of the pus is usually attended with very little danger, provided it is stopped at once, when the patient complains of a sense of pressure on the sternum, or has a strong inclination to cough, or when blood appears in the fluid.

Any of these symptoms indicate that more pressure on the delicate air-cells would be unsafe.

*Second.*—An operation in which a valvular opening is made with a scalpel, the wound being carefully closed after the pus has been evacuated. It is much less satisfactory in its results than those operations in which provision is made for free drainage of the cavity.

*Third.*—Chassaignac's operation for free drainage, which is done by making two openings in the chest, one above and the other below, and passing through them a perforated drainage tube.

This operation has fallen into disuse, as more recent methods have been found more successful.

*Fourth.*—An operation recommended by Fraentzel, in which the thorax is opened and a short metallic tube inserted, through which catheters may be introduced to wash out the cavity. This is one of the best operations for empyema, and there may be a few cases in which it is the very best, but the difficulties encountered in keeping the canula in position, and the amount of personal care from the physician which is demanded, make it less desirable, in the majority of cases, than the operation which I have performed for several years.

*Fifth.*—The operation which I like the best for its simplicity, convenience and good results, is made by puncturing the thoracic wall with a broad, flat trocar, through which two drainage tubes, or rather, a tube bent upon itself, is passed into the pleural sac. The canula of the trocar which I use is two inches in length, measuring one-half of an inch in its broadest diameter, and one-fourth of an inch in its shortest diameter. The drainage tube used is pure rubber tubing, with a caliber of one-eighth of an inch. The ends which are to hang in the pleural cavity are perforated, one for a distance of an inch, and the other a distance of about four inches, in order to facilitate perfect cleansing of the cavity.

I first make an incision in the skin one-half inch in length, through which the trocar is plunged into the pleural cavity. As the perforator is withdrawn, the pus spurts from the canula, but is immediately stopped by the thumb until the double tube can be picked up. The latter (the external ends of which have first been closed by a string) is then pushed in through the canula the required depth, usually four or five inches, and the canula withdrawn, when the tissues contract firmly on the rubber tube, so as to prevent either the entrance of air or the exit of pus.

In order to still farther protect the opening, the drainage tubes are now passed through a piece of sheet rubber three inches square, near the center of which, and within an eighth of an inch of each other, two small holes, one-twelfth inch in diameter, have been punched.

This sheet is carried down close to the surface of the chest, where it acts as a valve to prevent the entrance of air, should the tubes become loose. Then over each tube, by the aid of a canula, is carried a half-inch section of the same kind of tubing, through which two loops of stout cord have been passed.

These sections are carried down close to the chest, through the loops are passed adhesive straps to make them fast, and thus secure the drainage tubes, and over the dressing, close to the drainage tubes, is passed a bandage. The operation is then complete, and not more than an ounce of pus will have escaped, and not a particle of air will have entered.

The tubes may then be opened under water, and the pus allowed to escape, but in some cases, on account of shock, I have thought it best to delay washing out the cavity, or even allowing the pus to escape, until the next day.

With the tubes thus arranged, we have perfect control of the case, and if we desire it, all air can be excluded from the cavity for eight or ten days, but by the end of this time, the tissues usually retract about the tubes so that air is liable to enter; there is, however, no objection to this at this time, though earlier it is sometimes objectionable.

Usually, for the first three or four weeks after the operation, the cavity should be washed out daily with antiseptic solutions; later, stimulating washes of the sulphate or chloride of zinc, sulphate of copper, iodine, etc., must be used, if the cavity does not steadily decrease in size. I think it especially desirable to carefully watch the progress of healing after the cavity is reduced to a capacity of one or two ounces, for from this time on, healing is apt to be slow, and unless care is used, a troublesome fistula will remain.

